# EXPLORING THE WITHIN-PERSON RELATIONSHIP BETWEEN DAILY DISCRIMINATION AND DAILY STRESS REACTIVITY

**DOI:** 10.1093/geroni/igad104.2161

**Published:** 2023-12-21

**Authors:** Jessica Maras, Kate Leger

**Affiliations:** University of Kentucky, Lexington, Kentucky, United States; University of Kentucky, Lexington, Kentucky, United States

## Abstract

Experiencing discrimination has been associated with a variety of negative impacts such as poorer health and well-being. Some research has shown that those who experienced discrimination at an initial timepoint were more likely to have poorer health and well-being outcomes 20 years later through the mediating role of threat appraisals and stress reactivity. Research has demonstrated a link between experiencing discrimination and affective reactions to everyday stressful experiences, however, these associations have only been studied at the between-person level. For the current study, we examined the within-person relationship in daily stress reactivity in relation to experiencing discrimination on a daily basis over the course of 20 years. Participants (n = 1,315) completed waves 1 to 3 of the Midlife in the United States (MIDUS) survey with each wave about 10 years apart in which participants were asked to self-report their daily discrimination. Participants also completed the National Study of Daily Experiences (NSDE) which was a daily diary design over 8 consecutive days asking about their daily stress reactivity. Results found that during timepoints when people experienced greater than their average level of discrimination, they also reacted more negatively to daily stressful events (b = .002, 95% CI [0.001, 0.002]). These results are particularly striking because they show that within-person changes in reported discrimination are related to changes in affective responses to daily stressors. These findings suggest that experiencing discrimination is related to greater reactivity to daily stressors, which may lead to worse physical and mental health over time.

